# Long‐term treatment of cancer‐prone germline PTEN mutant mice with low‐dose rapamycin extends lifespan and delays tumour development

**DOI:** 10.1002/path.6009

**Published:** 2022-10-31

**Authors:** Priyanka Tibarewal, Victoria Rathbone, Georgia Constantinou, Wayne Pearce, Mahreen Adil, Zofia Varyova, Lisa Folkes, Alix Hampson, Gala Anastasia Electra Classen, Adriana Alves, Sara Carvalho, Cheryl L Scudamore, Bart Vanhaesebroeck

**Affiliations:** ^1^ Cancer Institute University College London London UK; ^2^ Oxford Institute of Radiation Oncology, Department of Oncology University of Oxford Oxford UK; ^3^ Exepathology Exmouth UK

**Keywords:** PTEN, PI 3‐kinase, PHTS, rapamycin, rare disease, cancer prevention, syndrome, hamartoma, mTORC1, drug, kinase inhibitor

## Abstract

*PTEN* is one of the most commonly inactivated tumour suppressor genes in sporadic cancer. Germline heterozygous *PTEN* gene alterations also underlie *PTEN* hamartoma tumour syndrome (PHTS), a rare human cancer‐predisposition condition. A key feature of systemic *PTEN* deregulation is the inability to adequately dampen PI3‐kinase (PI3K)/mTORC1 signalling. PI3K/mTORC1 pathway inhibitors such as rapamycin are therefore expected to neutralise the impact of *PTEN* loss, rendering this a more druggable context compared with those of other tumour suppressor pathways such as loss of *TP53*. However, this has not been explored in cancer prevention in a model of germline cancer predisposition, such as PHTS. Clinical trials of short‐term treatment with rapamycin have recently been initiated for PHTS, focusing on cognition and colon polyposis. Here, we administered a low dose of rapamycin from the age of 6 weeks onwards to mice with heterozygous germline *Pten* loss, a mouse model that recapitulates most characteristics of human PHTS. Rapamycin was well tolerated and led to a highly significant improvement of survival in both male and female mice. This was accompanied by a delay in, but not full blockade of, the development of a range of proliferative lesions, including gastro‐intestinal and thyroid tumours and endometrial hyperplasia, with no impact on mammary and prostate tumours, and no effect on brain overgrowth. Our data indicate that rapamycin may have cancer prevention potential in human PHTS. This might also be the case for sporadic cancers in which genetic PI3K pathway activation is an early event in tumour development, such as endometrial cancer and some breast cancers. To the best of our knowledge, this is the first report of a long‐term treatment of a germline cancer predisposition model with a PI3K/mTOR pathway inhibitor. © 2022 The Authors. *The Journal of Pathology* published by John Wiley & Sons Ltd on behalf of The Pathological Society of Great Britain and Ireland.

## Introduction

The class I PI3Ks and their downstream protein kinase effectors AKT and mTORC1 are drivers of cancer development and progression. This signalling pathway is kept in check by the tumour suppressor PTEN, which dephosphorylates the lipids produced by these PI3Ks [1, 2, 3, 4, 5].

*PTEN* is frequently somatically inactivated in a broad range of cancers [6, 7]. Germline heterozygous *PTEN* inactivation in humans manifests as a complex multi‐organ disorder known as *PTEN* hamartoma tumour syndrome (PHTS) [8]. These individuals often present with hamartomatous skin lesions and benign gastro‐intestinal (GI) polyps, with a predisposition for certain types of cancer, brain overgrowth, and autism spectrum disorder (ASD). There are currently no approved targeted treatments for PHTS, with specific symptoms and cancers being treated as and when they occur [9]. Given the availability of multiple PI3K pathway inhibitors [10], the possibility exists that these could be used in preventative or therapeutic settings in PHTS.

Rapamycin and rapalogs (such as RAD001) are allosteric mTORC1 inhibitors [11]. They act by binding to FKBP12, a key component of the mTORC1 complex, hereby inhibiting the activity of this complex and disrupting the activation of downstream effectors such as S6 kinase and 4E‐BP1 which are required for key cellular processes such as mRNA translation, cell division, and cell proliferation. These drugs have been approved for the treatment of specific cancers such as renal cell carcinoma [12] and neuroendocrine tumours of pancreatic origin [13]. Rapamycin is also used as an immunosuppressant in kidney transplantation and for management of certain rare diseases such as tuberous sclerosis (TS) and lymphangioleiomyomatosis [11, 14, 15]. Rapamycin also prolongs lifespan in diverse species including mammals, and delays the onset of age‐related disease and the development of lethal neoplasia [16] (reviewed in refs 17, 18, 19, 20). However, the impact of life‐long treatment with a low dose of rapamycin in a germline cancer predisposition context has not been tested to date. Here, we explored the effect of life‐long treatment with a low dose of rapamycin in PHTS using heterozygous germline *Pten* knockout mice (*Pten* ^*+/−*^). These mice phenocopy human PHTS, in that they present with macrocephaly and tumours of the thyroid, GI tract, mammary, and endometrium (reviewed in refs 21 and 22). Our study is timely, given that mTORC1 inhibitors have recently entered clinical trials for the management of PHTS (NCT02991807 and NCT04094675).

## Materials and methods

### Mice and rapamycin treatment

*Pten* ^*+/−*^ mice were from Ramon Parsons [23]. Sv129 mice were purchased from Charles Rivers Laboratories (Saffron Waldon, UK) or Envigo (Bicester, UK) and crossed with C57Bl/6J *Pten* ^*+/−*^ mice to obtain F1 mixed background mice, which were used for all experiments. All mice were maintained at University College London in accordance with The Animals (Scientific Procedures) Act 1986 Amendment Regulations 2012. Rapamycin encapsulated in Eudragit‐S100 (eRapa) was from Rapamycin Holdings (San Antonio, TX, USA) and was incorporated at 14 ppm (eRapa diet) in the mouse diet by Test Diet Ltd (Richmond, IN, USA), with an equivalent amount of Eudragit‐S100 incorporated in mouse chow as the control diet. *Pten* ^*+/−*^ mice and littermate wild‐type *Pten* ^+/+^ control mice were switched to either the control or the eRapa diet when reaching the age of 6 weeks (or 4 weeks when indicated). Once put on a new diet, mice were weighed twice a week. They were monitored and euthanised if they presented with signs of illness or had palpable lumps with a total surface area of ≥1.4 cm^2^. In the lifetime studies, animals showing no clinical signs were euthanised at the end of the study (600 days for females and 730 days for males).

Other methodology is described in Supplementary materials and methods.

## Results

### Rapamycin dose selection

The clinically effective plasma concentration of rapamycin in TS patients is 2–10 ng/ml [24, 25], with a similar dose exposure aimed at ongoing human PHTS trials. Following placement of mice on an eRapa diet (containing 14 ppm eRapa), Wilkinson *et al* showed previously that this resulted in serum levels of rapamycin of 13.4 ± 2.6 ng/ml [16]. This dose of rapamycin has also been shown to extend lifespan and to slow multiple parameters of ageing in mice [16, 26, 27]. We therefore decided to use this eRapa diet for our studies. Diet containing the encapsulating polymer alone (control diet) was fed to control groups.

**Figure 1:**
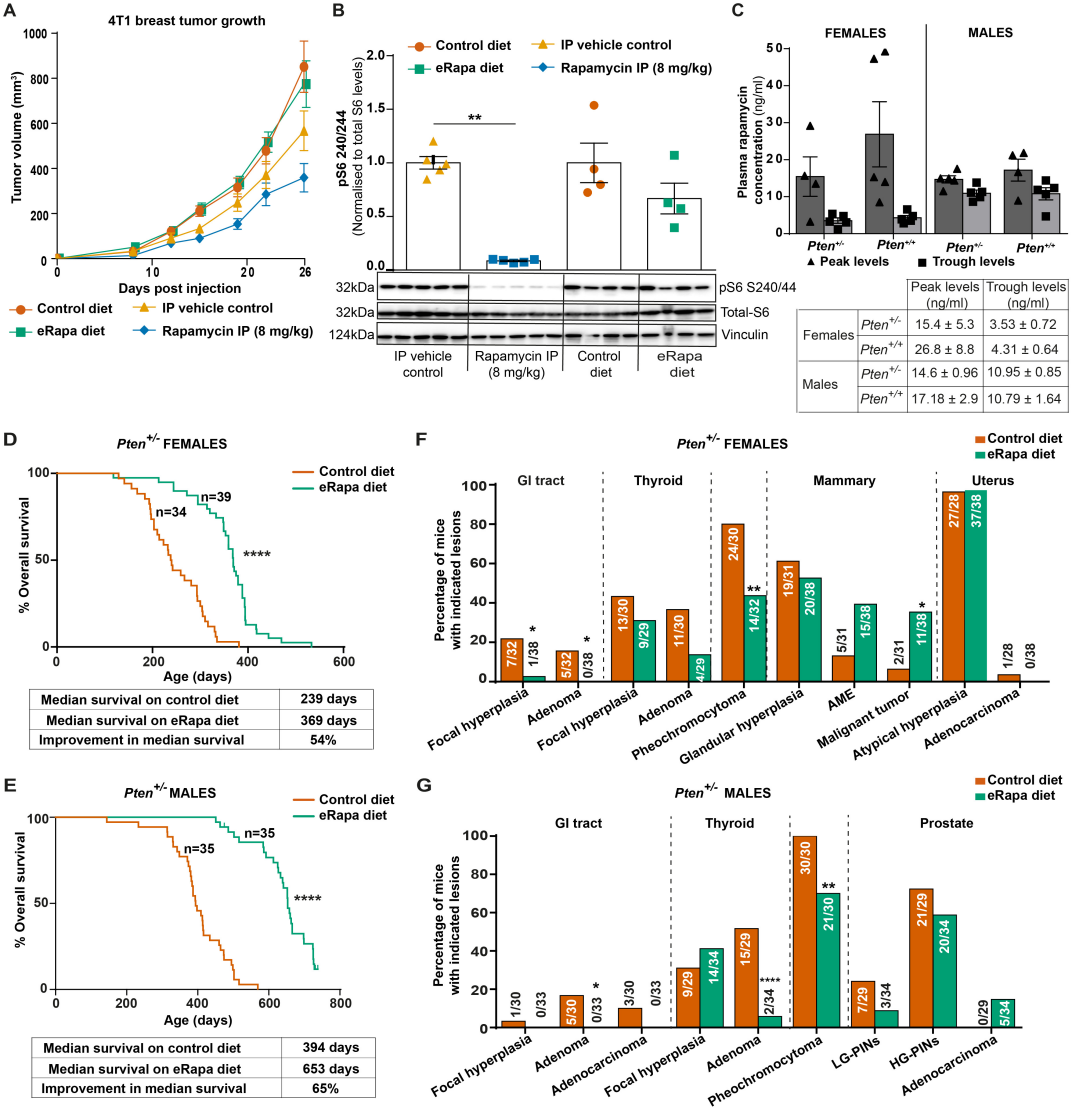
Long‐term treatment with low dose rapamycin improves overall survival in *Pten* ^*+/−*^ mice. (A) Effect of rapamycin on 4T1 tumour growth. BALB/c mice were injected with 4T1 mammary cancer cells and treated with rapamycin IP (8 mg/kg) (once a day – 5 days on/2 days off) or with the eRapa diet. Mice injected with vehicle (IP vehicle control) or on the control diet were used as controls. Tumours were measured using calipers. (B) Immunoblot analysis of protein extracts from the livers of mice treated as indicated. Statistical analysis was performed using a Mann–Whitney test. **p < 0.01. (C) Plasma rapamycin concentrations in *Pten* ^*+/−*^ and *Pten* ^*+/+*^ mice on a mixed (C57BL/6J × Sv129) background fed the control or eRapa diet from the age of 6 weeks. Blood samples were taken at 6 months of age (18 weeks after diet switch). The table shows the levels of rapamycin observed at peak levels (blood sample taken at 9 am) and trough levels (blood sample taken at 2 pm, after 6 h of starvation). (D) Kaplan–Meier survival curves for female *Pten* ^*+/−*^ mice on a mixed (C57BL/6J × Sv129) background fed the control or eRapa diet from the age of 6 weeks. Mice were monitored and were euthanised for welfare reasons (masses with a combined size of ≥1.4 cm^2^ surface area or ill health) or at the end of the study. (E) Same as in D, but for male mice. Statistical analysis in D and E was performed using the log‐rank (Mantel–Cox) test and Gehan–Breslow–Wilcoxon test. ****p < 0.0001. (F, G) Histopathological analysis was carried out on all mice to assess the incidence of specific tumour types in female (F) and male (G) *Pten* ^*+/−*^ mice fed the control or eRapa diet from the age of 6 weeks onwards. Statistical analysis was performed using Fisher's exact test. *p < 0.05; **p < 0.01; ***p < 0.001; ****p < 0.0001.

Before embarking on a life‐long exposure experiment, we explored whether the eRapa diet had an acute, direct anti‐cancer effect using transplanted 4T1 syngeneic mammary tumours, which have been previously shown to regress upon administration of high‐dose (4 mg/kg body weight) rapamycin [28]. BALB/c mice subcutaneously injected with 4T1 cells were given an i.p. injection of rapamycin (8 mg/kg body weight) or fed the eRapa diet. Based on published data using these doses [29], the expected peak and trough levels of rapamycin are much higher upon i.p. injection (reported peak levels: 1842 ± 130 ng/ml; 24 h trough levels: 45.1 ± 2.4 ng/ml [29]) compared with the peak levels in mice fed the eRapa diet (12.8 ± 2 ng/ml). In line with expectations, the i.p. dose of 8 mg/kg slowed tumour growth (Figure 1A). This could be attributed to its ability to almost completely suppress mTORC1 signalling, as shown by the >90% reduction in phosphorylation of the S6 ribosomal protein (on S240/244) in the livers of these mice (Figure 1B). In contrast, the eRapa diet did not slow down 4T1 tumour growth, most likely due to its inability to completely suppress mTORC1 signalling, as seen in the livers of these mice (30% reduction of S240/244 phosphorylation; Figure 1B). However, such partial suppression of signalling by the eRapa diet might be sufficient to normalise the modest mTORC1 pathway activation that can result from heterozygous PTEN loss and this dose was therefore used for our long‐term studies.

### Long‐term treatment with rapamycin significantly improves the overall survival of *Pten* ^*+/−*^ mice

To investigate the effect of long‐term treatment of rapamycin on overall survival and tumour burden, 6‐week‐old *Pten* ^*+/−*^ and *Pten* ^+/+^ mice were switched to the eRapa diet or control diet and monitored for tumours and illness.

At 6 months of age (18 weeks post‐diet switch), the eRapa diet led to peak plasma levels of rapamycin of ~14–27 ng/ml and trough levels (after 6 h of starvation) of ~3–11 ng/ml (Figure 1C), consistent with what was reported earlier for other mouse strains and within the range we had aimed for.

Long‐term treatment of *Pten* ^*+/−*^ mice with the eRapa diet significantly improved the overall survival of both female and male mice (54% and 65% improvement in median survival, respectively; *p* < 0.0001; Figure 1D,E). On both the control and the eRapa diet, the main reason for culling mice was the presence of palpable lumps (enlarged lymph nodes or mammary tumours) that would otherwise exceed volumes allowed under UK Animal Welfare regulations.

Histopathological analysis of selected tissues from control and rapamycin‐treated mice revealed very similar morphologies of all tissues analysed, with overall histopathology data summarised in Figure 1F,G and Table 1. The cause of illness leading to the humane endpoint euthanasia is summarised in supplementary material, Tables S2 and S3. Below, we describe the impact of rapamycin on tumour development in individual tissues.

**Table 1:** Histopathological analysis of tissues from control and rapamycin‐treated mice.

		Female mice		Male mice	
Tissue	Lesion type	Control diet	Rapamycin diet	Control diet	Rapamycin diet
GI tract	Focal hyperplasia	22% (7/32)	3% (1/38)	3% (1/30)	0% (0/33)
	Adenoma	16% (5/32)	0% (0/38)	17% (5/30)	0% (0/33)
	Adenocarcinoma	0% (0/32)	0% (0/38)	10% (3/30)	0% (0/33)
Thyroid	Focal hyperplasia	43% (13/30)	31% (9/29)	31% (9/29)	41% (14/34)
	Follicular adenoma	37% (11/30)	14% (4/29)	52% (15/29)	6% (2/34)
Adrenal	Phaeochromocytoma	80% (24/30)	44% (14/32)	100% (30/30)	70% (21/30)
Mammary	Glandular hyperplasia	61% (19/31)	52% (20/38)		
	Adenomyoepithelioma (AME)	13% (5/31)	39% (15/38)		
	Malignant tumour	6% (2/31)	35% (11/38)		
Uterus	Atypical hyperplasia	97% (27/28)	97% (37/38)		
	Adenocarcinoma	4% (1/28)	0% (0/38)		
Prostate	LG‐PINs			24% (7/29)	9% (3/34)
	HG‐PINs			72% (21/29)	59% (20/34)
	Adenocarcinoma			0% (0/29)	15% (5/34)
Lymph nodes	Lymphoid hyperplasia (average score)	3.6 (*n* = 33)	2.8 (*n* = 36)	3.46 (*n* = 32)	2.15 (*n* = 33)
Spleen	Extramedullary haematopoiesis (EMH) (average score)	3.15 (*n* = 27)	3.66 (*n* = 36)	2.87 (*n* = 32)	2 (*n* = 34)

### Rapamycin slowed GI and thyroid tumour development in *Pten* ^*+/−*^ mice

**Figure 2:**
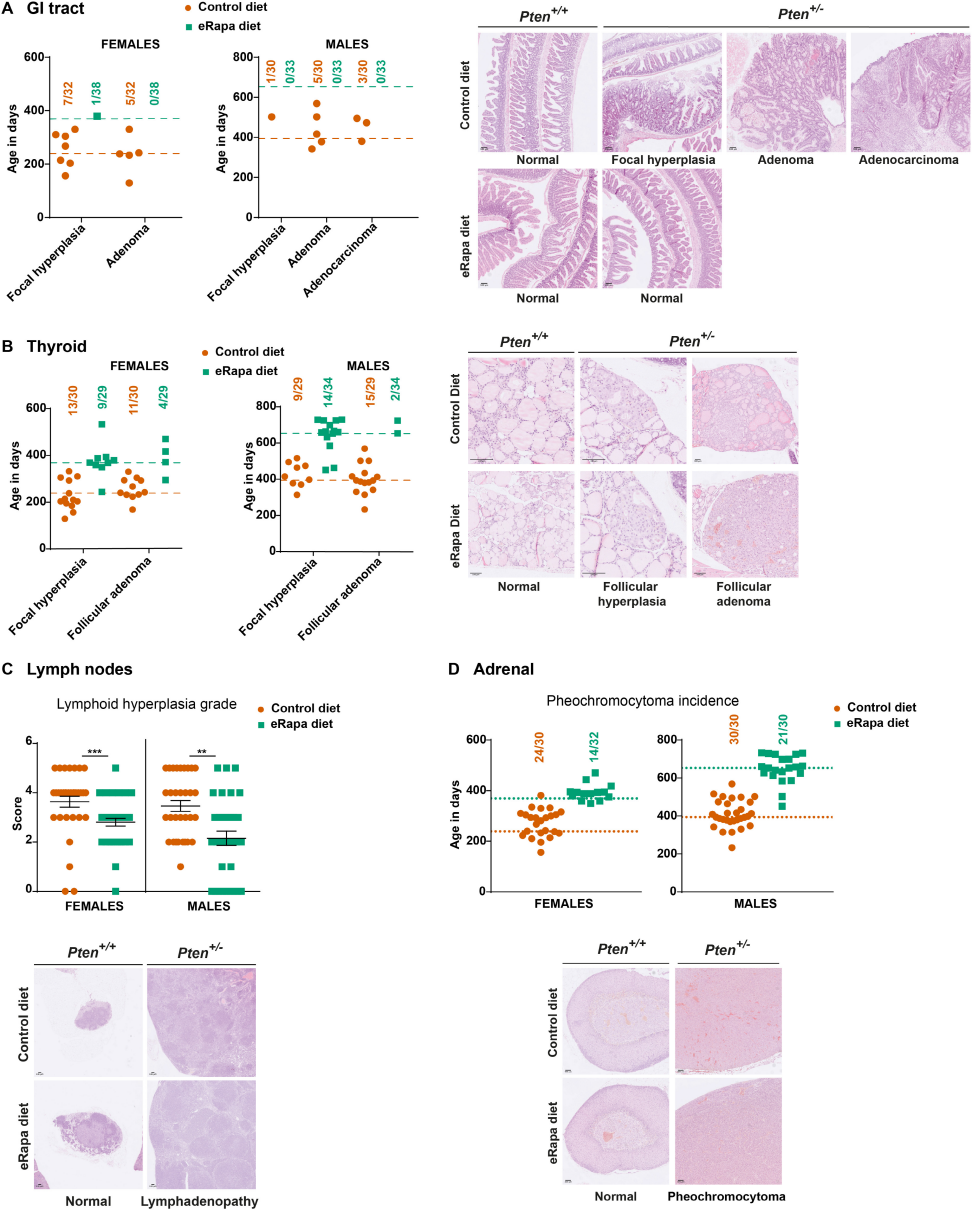
Low dose rapamycin is effective at delaying GI tract, thyroid, and adrenal tumours, and lymphoid hyperplasia. *Pten* ^*+/−*^ mice on a mixed (C57BL/6J × Sv129) background were fed the control or eRapa diet from the age of 6 weeks onwards. Mice were euthanised for welfare reasons (masses with a combined size of ≥1.4 cm^2^ surface area or ill health) or at the end of the study, and histopathological analysis was performed. (A) Incidence of GI tumours and distribution of age of mice presenting with the indicated tumour in female (left panel) and male (middle panel) mice. Dotted lines show the median survival age of mice on the control diet (orange) or the eRapa diet (green). Representative photomicrographs of H&E‐stained sections showing GI tract histopathology in *Pten* ^*+/−*^ and *Pten* ^+/+^ mice on the control or eRapa diet (right panel). (B) Incidence of thyroid tumours and distribution of age of mice presenting with the indicated tumours in female (left panel) and male (middle panel) mice. Dotted lines show the median survival age of mice on the control diet (orange) or the eRapa diet (green). Representative photomicrographs of H&E‐stained sections showing thyroid histopathology in *Pten* ^*+/−*^and *Pten* ^+/+^ mice on the control or eRapa diet (right panel). (C) Grade of lymphoid hyperplasia (top panel) and representative photomicrographs of H&E‐stained sections showing lymph node histopathology in *Pten* ^*+/−*^and *Pten* ^+/+^ mice on the control or eRapa diet (bottom panel). (D) Incidence and distribution of age of mice presenting with phaeochromocytoma in female (top left panel) and male (top right panel) mice. Dotted lines show the median survival age of mice on the control diet (orange) or the eRapa diet (green). Representative photomicrographs of H&E‐stained sections showing the histopathology of the adrenal glands in *Pten* ^*+/−*^ and *Pten* ^+/+^ mice on the control or eRapa diet (bottom panel). Statistical analysis was performed using Mann–Whitney tests. *p < 0.05; **p < 0.01; ***p < 0.001; ****p < 0.0001. Scale bars in A, B, C and D represent 100 μm.

Long‐term rapamycin treatment significantly reduced the incidence of GI adenomas in both female and male mice and focal hyperplasia in females, A reduction in GI adenocarcinomas was also seen in eRapa diet‐fed males (Figures 1F,G and 2A; Table 1).

Rapamycin also reduced the incidence of thyroid focal hyperplasia and adenomas in female mice (Figures 1F and 2B; Table 1). In the rapamycin‐treated male mice, while the incidence of thyroid adenomas was significantly reduced, the incidence of hyperplasia was slightly higher (Figures 1G and 2B; Table 1). Since the eRapa‐treated mice were older than mice on the control diet, this suggests that rapamycin treatment had considerably slowed the progression of thyroid lesions.

### Rapamycin reduced lymphoid overgrowth in *Pten* ^*+/−*^ mice

In the long‐term study, although all *Pten* ^*+/−*^ mice both on the control and on the eRapa diet presented with hyperplasia of peripheral lymph nodes (cervical, brachial, axillary, and inguinal lymph nodes), rapamycin treatment significantly reduced the grade of these lesions (as assessed by a tissue score; Figure 2C). A similar reduction was seen in an additional cohort where 6‐week‐old mice were fed the control or eRapa diet and sacrificed at 6 months of age (referred to as Timed Study I) (supplementary material, Figure S1A).

### Rapamycin reduced the incidence of phaeochromocytomas in *Pten* ^*+/−*^ mice

Although there are no known links between PTEN and the pathogenesis of adrenal phaeochromocytomas in humans, *Pten* ^*+/−*^ mice present with a high incidence of phaeochromocytomas [21, 30], which was significantly reduced in both female and male mice on the eRapa diet (Figures 1F,G and 2D; Table 1).

### Rapamycin treatment delayed the development of endometrial lesions in female *Pten* ^*+/−*^ mice

**Figure 3:**
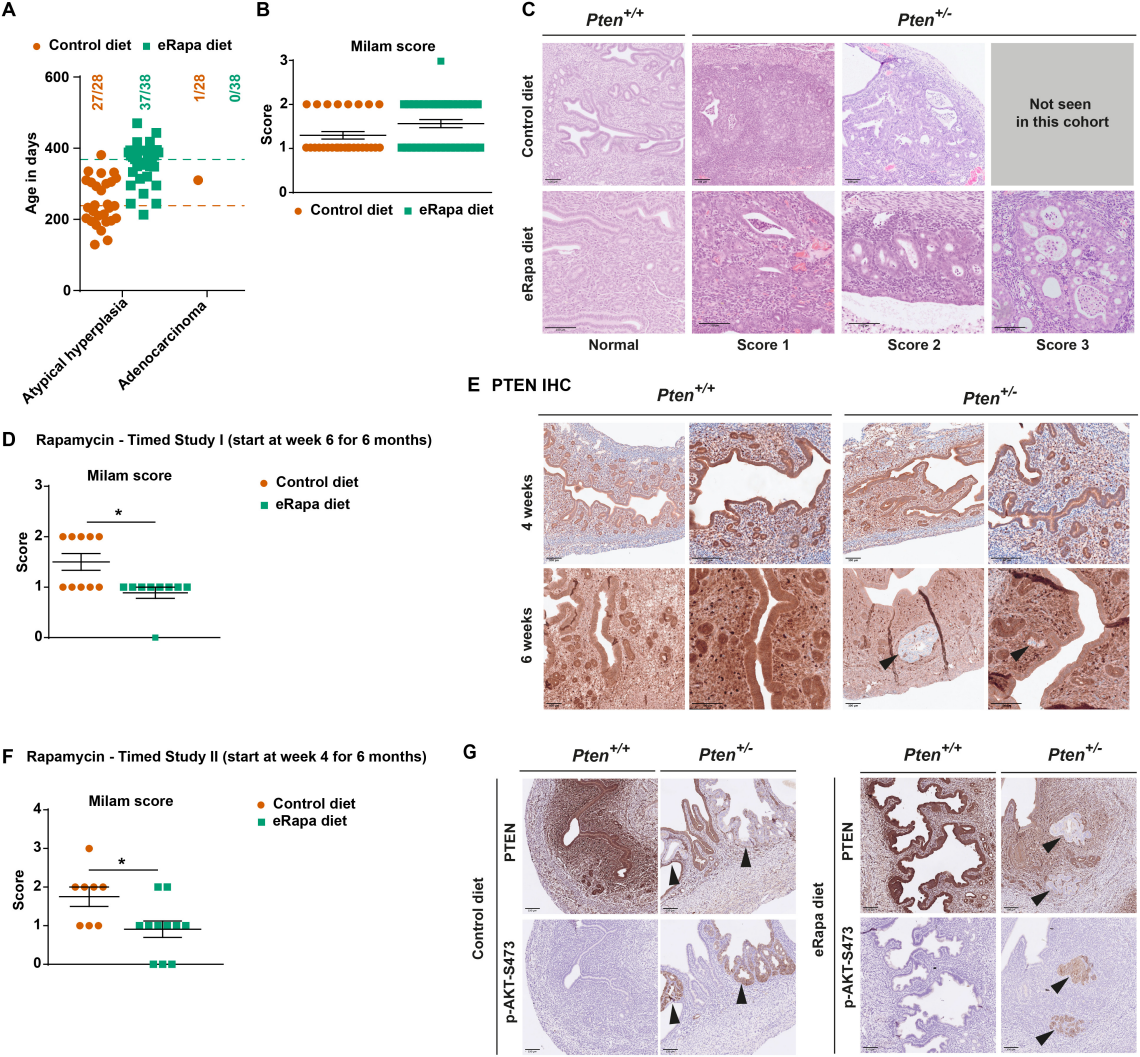
Low dose rapamycin is effective at delaying endometrial hyperplasia. *Pten* ^*+/−*^ mice on a mixed (C57BL/6J × Sv129) background were fed the control or eRapa diet from the age of 6 weeks onwards. Mice were euthanised for welfare reasons (masses with a combined size of ≥1.4 cm^2^ surface area or ill health) or at the end of the study, and histopathological analysis was performed. (A) Incidence and age distribution of endometrial proliferative lesions in mice presenting with the indicated lesions in female *Pten* ^*+/−*^ mice on the control or eRapa diet. Dotted lines show the median survival age of mice on the control diet (orange) or the eRapa diet (green). (B) Grade of atypical hyperplasia seen in mice in the life‐long study in A, determined using the Milam scoring system (described in supplementary material, Supplementary materials and methods). (C) Representative photomicrographs of H&E‐stained sections showing the uterine histopathology in *Pten* ^*+/−*^ and *Pten* ^+/+^ mice on the control or eRapa diet. (D) Rapamycin Timed Study I: *Pten* ^*+/−*^ mice at 6 weeks of age were switched to the control or eRapa diet and sacrificed when 6 months old, followed by analysis of the uteri by H&E staining and Milam scoring. (E) Representative examples of IHC performed on uteri from 4‐ and 6‐week‐old female *Pten* ^+/+^ and *Pten* ^*+/−*^ mice showing loss of PTEN immunoreactivity in endometrial glands of *Pten* ^*+/−*^ mice at 6 weeks (indicated by black arrowheads) but not at 4 weeks of age. Additional images are shown in supplementary material, Figure S2. (F) Rapamycin Timed Study II: *Pten* ^*+/−*^ mice at 4 weeks of age were switched to the control or eRapa diet and sacrificed at the age of 6 months, followed by analysis of uteri by H&E staining and Milam scoring. (G) Representative examples of IHC performed on uteri from female mice from Timed Study II showing loss of PTEN immunoreactivity in endometrial glands of *Pten* ^*+/−*^ mice on the control or eRapa diet (indicated by black arrowheads), with a corresponding increase in pAKT‐S473 immunoreactivity within the same glands (indicated by black arrowheads). Additional images are shown in supplementary material, Figure S3. Statistical analysis was performed using Mann–Whitney tests. *p < 0.05; **p < 0.01; ***p < 0.001; ****p < 0.0001. Scale bars in C, E and G represent 100 μm.

In human sporadic cancer, endometrial tumours have the highest frequency of *PTEN* mutations [31]. Female *Pten* ^*+/−*^ mice also have a very high incidence of endometrial lesions, with almost 100% of the mice showing atypical endometrial hyperplasia by the age of 6 months, a phenotype also observed in all mice on the eRapa diet (adenocarcinoma was found only in one mouse on the control diet) (Figures 1F and 3A; Table 1). Using a scoring system for mouse endometrial lesions developed by Milam *et al* [32], we found that there was no significant difference in the grade of the endometrial lesions between the control and the rapamycin‐treated group (Figure 3B). However, the interpretation of this observation is complicated by the fact that the rapamycin‐treated mice lived substantially longer, possibly allowing time for these cancers to develop.

To determine if there was a delay in progression of the endometrial lesions, we performed histopathological analysis of the uteri of mice from Timed Study I (6‐week‐old mice were fed the control or eRapa diet and sacrificed at 6 months of age), which revealed significantly lower‐grade endometrial lesions in rapamycin‐treated mice, indicating that rapamycin slows down but does not prevent the development of endometrial lesions (Figure 3D).

Loss of PTEN protein expression is one of the proposed mechanisms of the progression of endometrial tumours in *Pten* ^*+/−*^ mice [33]. Immunohistochemical (IHC) analysis revealed loss of PTEN protein immunoreactivity in some endometrial glands in 9/14 *Pten* ^*+/−*^ mice as early as 6 weeks of age but not in 4‐week‐old *Pten* ^*+/−*^ mice (Figure 3E and supplementary material, Figure S2). It was therefore of interest to explore if starting rapamycin treatment at 4 weeks of age would suppress the loss of PTEN protein expression and completely prevent endometrial tumour development. However, switching mice to the eRapa diet at 4 weeks and analysis at 6 months of age (referred to as Timed Study II) revealed that although rapamycin‐treated mice presented with a lower grade of the endometrial lesions, rapamycin treatment did not completely block the development of endometrial hyperplasia (Figure 3F). The uteri of 6‐month‐old mice also presented with areas of no PTEN expression and concomitant pAKT‐S473 increase (Figure 3G and supplementary material, Figure S3), suggesting that rapamycin treatment did not prevent the loss of protein expression of the wild‐type copy of PTEN.

### Rapamycin treatment did not prevent the occurrence of mammary tumours

Female *Pten* ^*+/−*^ mice have been reported to develop malignant mammary tumours around 7 months of age, with 100% of the mice showing mammary tumours by the age of 15 months [21, 34]. In our study, mice on the control diet did not reach the age at which the mammary tumours would have developed, given that the majority of the female mice develop enlarged lymph nodes around 5–6 months, which upon reaching 1.4 cm^2^ require euthanasia under UK Animal Welfare regulations (supplementary material, Table S1).

**Figure 4:**
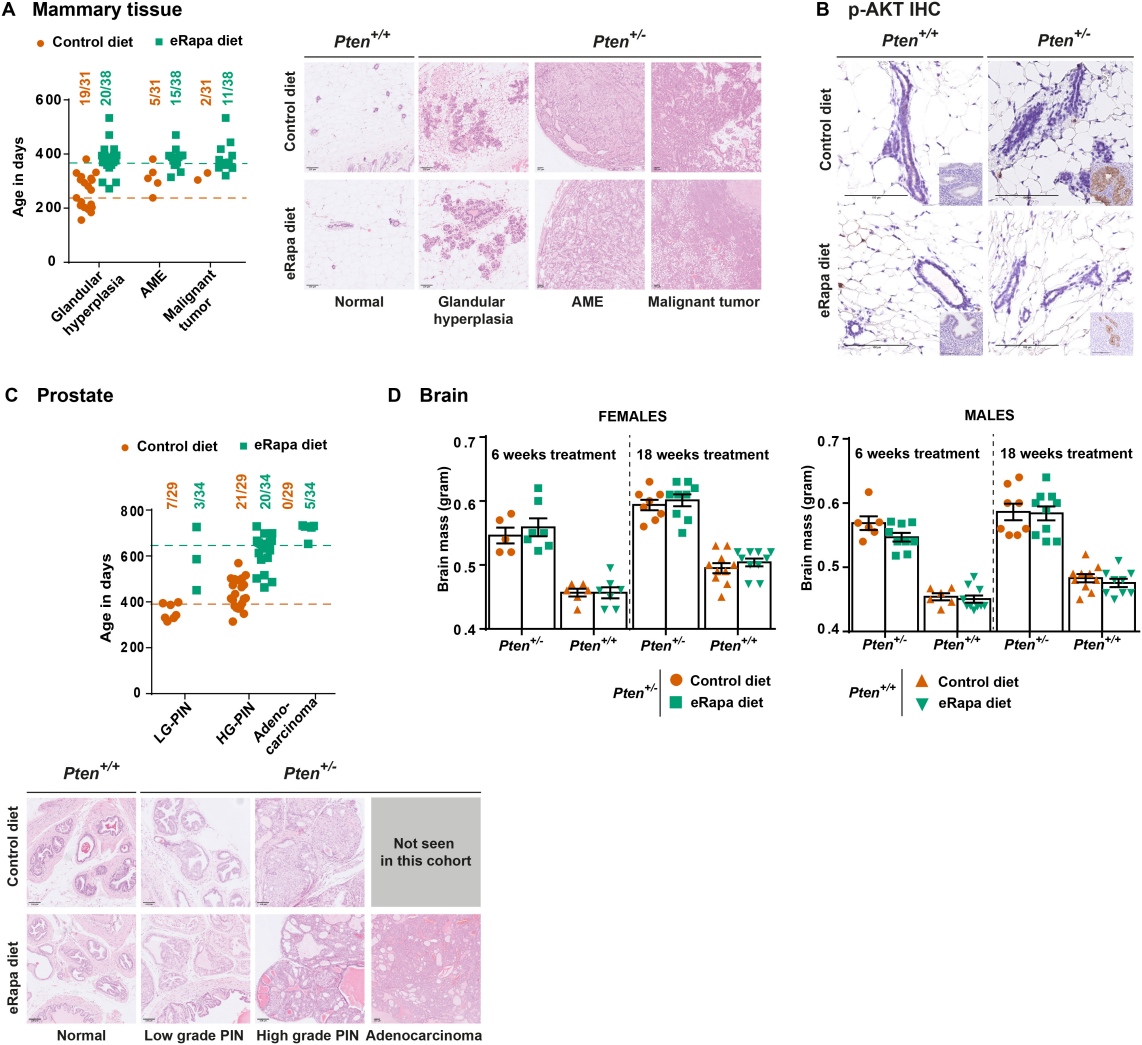
Low dose Rapamycin is not effective at delaying prostate and mammary tumours and does not rescue macrocephaly. *Pten* ^*+/−*^ mice on a mixed (C57BL/6J × Sv129) background were fed the control or eRapa diet from the age of 6 weeks onwards. Mice were euthanised for welfare reasons (masses with a combined size of ≥1.4 cm^2^ surface area or ill health) or at the end of the study, and histopathological analysis was performed. (A) Incidence of mammary tumours and age distribution of female *Pten* ^*+/−*^ mice presenting with the indicated lesions (left panel). Dotted lines show the median survival age of mice on the control diet (orange) or the eRapa diet (green). Representative photomicrographs showing the histological characteristics of mammary tumours (right panel). (B) IHC analysis of pAKT‐S473 in mammary tissues of 6‐month‐old female mice switched to the control or eRapa diet at 4 weeks of age. No signal for pAKT‐S473 was observed in mammary tissues of mice on the control or eRapa diet. Staining for pAKT‐S473 in endometrial tissue of the same mouse processed on the same slide is shown as an inset. Note the positive pAKT‐S473 staining in *Pten* ^*+/−*^ endometrium but absence of the staining in *Pten* ^+/+^ endometrium. Additional images are shown in supplementary material, Figure S4. (C) Incidence of prostate tumours in male mice (top panel). Dotted lines show the median survival age of mice on the control diet (orange) or the eRapa diet (green). Representative photomicrographs showing the histological characteristics of prostate tumours (bottom panel). (D) Brain weight of *Pten* ^*+/−*^ and *Pten* ^+/+^ littermate mice fed the control or eRapa diet from 6 weeks of age for 6 or 18 weeks. Statistical analysis was performed using Mann–Whitney tests. Scale bars in A, B and C represent 100 μm.

At the time of sacrifice a substantial fraction of control *Pten* ^*+/−*^ mice had hyperplasia of the mammary glands (19/31), with 5/31 having developed adenomyoepithelioma (AME), a benign growth, with only 2/31 showing malignant mammary tumours (Figures 1F and 4A; Table 1). At the end of the study, rapamycin‐treated mice had a higher incidence of AME (15/38) and malignant mammary tumours (11/38) than the control group (Figures 1F and 4A; Table 1).

This apparent increased mammary tumour burden in long‐term rapamycin‐treated mice is counterintuitive. We next investigated whether this observation could be related to so‐called feedback activation of the PI3K/mTORC1 pathway upon rapamycin treatment. These are adaptive signalling pathways which neutralise the impact of pharmacological mTORC1 inhibition, leading to a rebound signalling in this pathway [4, 35, 36, 37, 38]. These compensatory signalling loops can outweigh and outlast the impact of mTORC1 inhibition, for example by inducing the expression of tyrosine kinase receptors [36]. In order to gain insight into this, we analysed histology and signalling in mammary tissue of mice from Timed Study II. None of the mice in the control and rapamycin‐treated groups (*n* = 10 in each group) had mammary tumours at 6 months, with the exception of one mouse in the control group which had AME (supplementary material, Figure S1B). The level of IHC‐based pAKT‐S473 in tissues from both the control and the treated group at 6 months was very low and not enhanced in drug‐treated mice compared with untreated mice (Figure 4B and supplementary material, Figure S4). This indicates that rapamycin treatment had not induced mTORC1/AKT feedback activation in the mammary gland and thus did not promote mammary tumour development.

Taken together, the higher incidence of mammary tumours in rapamycin‐treated mice at the end of life is most likely a consequence of these mice living longer, allowing time for malignant mammary tumours in the drug‐treated group to develop, with rapamycin not able to suppress the development of these tumours.

### Rapamycin did not slow the development of prostate tumours in *Pten* ^*+/−*^ male mice

Although male PHTS patients do not show enhanced predisposition for prostate cancer, loss of PTEN expression is common in somatic prostate cancer [39]. *Pten* ^*+/−*^ mice are known to develop prostatic intraepithelial neoplasias (PINs) but almost never present with advanced tumours such as prostate adenocarcinomas (CaPs) (Figures 1G and 4C; Table 1).

In our study, while rapamycin treatment reduced the incidence of both low‐ and high‐grade PINs (Figures 1G and 4C; Table 1), 5/34 mice in the treatment group presented with CaPs (Figure 4C). As was the case with the mammary tumours, it was possible that detection of advanced tumours in the treated group was possible because the mice lived longer, with rapamycin unable to suppress prostate tumour development.

To rule out prostate tumour promotion by PI3K/mTORC1 feedback activation in eRapa‐fed mice, we set up a cohort of male mice which were fed the eRapa or control diet from 6 weeks to 6 months of age (*n* = 10 in each group). In this study, none of the mice in the control and drug‐treated groups presented with prostate lesions at 6 months. This time point is most likely too early for prostate tumours to develop, but the observation that the treated group did not even present with low‐grade lesions indicates that rapamycin does not promote prostate cancer development in *Pten* ^*+/−*^ mice.

### Rapamycin did not rescue macrocephaly in *Pten* ^*+/−*^ mice

PHTS patients often present with macrocephaly resulting from brain overgrowth [40]. Brain enlargement is also observed in *Pten* ^*+/−*^ mice [21, 41], including in our mouse cohort (Figure 4D). Treatment with rapamycin did not affect brain weight after 6 or 18 weeks of treatment of wild‐type and *Pten* ^*+/−*^ mice (Figure 4D). Rapamycin has been shown to reduce the increased brain mass in neuron‐specific, homozygous *Pten* knockout mice [42] but this was following i.p. injection of rapamycin at 10 mg/kg for 4–6 weeks, a substantially higher dose than in our study. It is most likely that our eRapa diet did not lead to sufficient drug exposure in the brain.

### Long‐term treatment with the eRapa diet was well tolerated but led to mild insulin resistance in *Pten* ^*+/−*^ mice

Overall, the eRapa diet did not noticeably affect the behaviour and well‐being of wild‐type or *Pten* ^*+/−*^ mice. To start to gain insight into the tolerability of long‐term rapamycin treatment, we tested a range of metabolic parameters in our mice.

Long‐term rapamycin treatment has been reported to dampen normal weight gain in mice, due to a reduction in adiposity [43]. In line with this, rapamycin‐treated wild‐type mice had a lower body weight compared with control diet mice, an effect most prominent in female mice (supplementary material, Figure S5). In *Pten* ^*+/−*^ mice, rapamycin treatment led to increased weight gain compared with control mice, an effect most significant in male mice (supplementary material, Figure S5).

Chronic rapamycin treatment has also been shown to affect glucose and fat metabolism in mice [44, 45], with an eRapa diet at 14 ppm leading to insulin resistance and glucose intolerance in some wild‐type mouse strains [45]. We therefore performed insulin tolerance tests (ITTs) and glucose tolerance tests (GTTs) on these mice.

**Figure 5:**
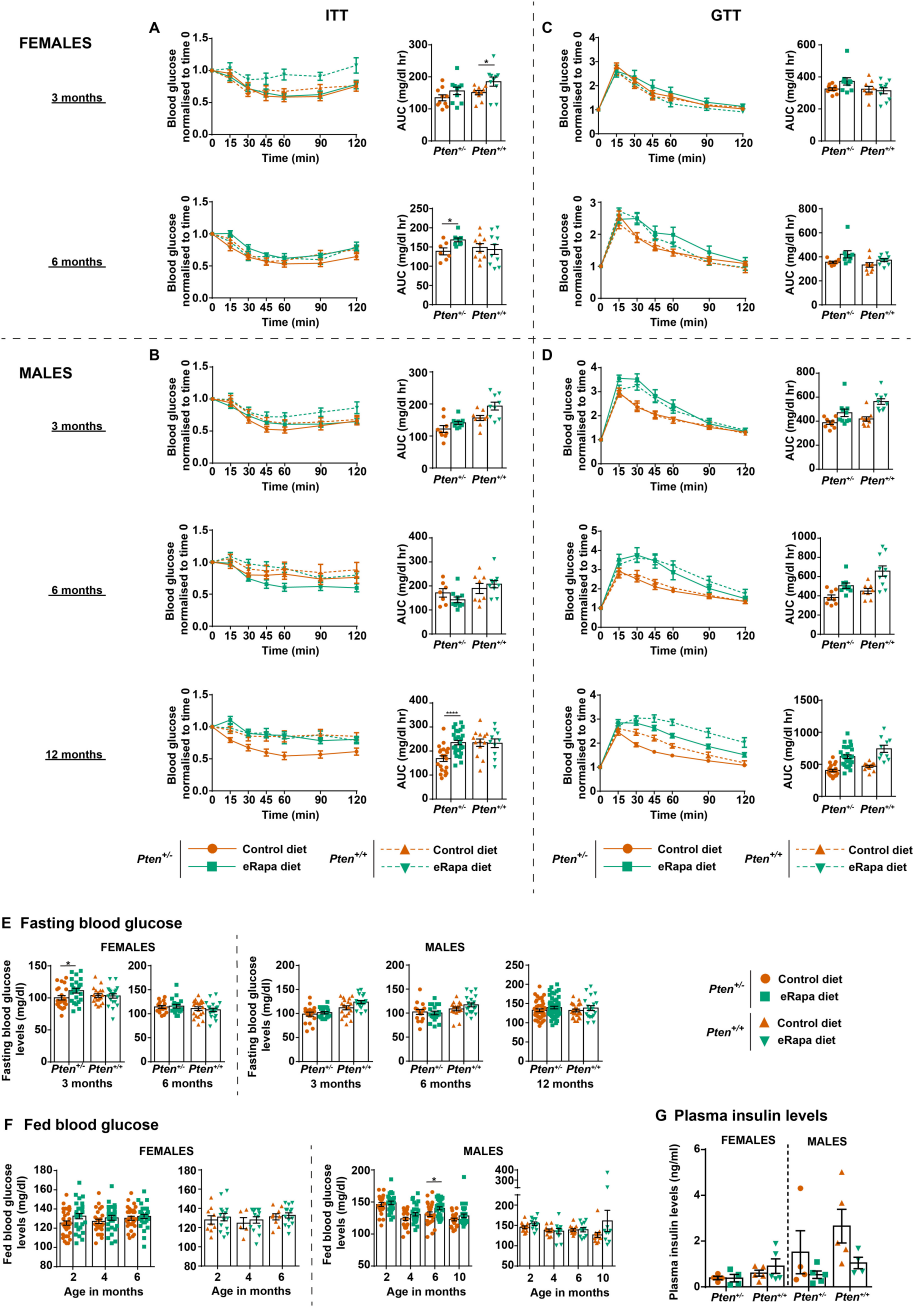
Effects of low dose rapamycin treatment on glucose metabolism. *Pten* ^*+/−*^ mice and littermate *Pten* ^+/+^ mice were fed the control or eRapa diet from 6 weeks of age. (A, B) ITT and (C, D) GTT assays performed at the indicated time points. Bar graphs show measurements of the area under the curve (AUC). (E) Mice were starved for 6 h and their fasting blood glucose was measured. (F) Fed blood glucose measurements, taken at 9 am at the indicated time points. (G) Insulin levels in blood samples harvested at 9 am from 6‐month‐old mice. Statistical analysis was performed using Mann–Whitney tests. *p < 0.05; **p < 0.01; ***p < 0.001; ****p < 0.0001.

In wild‐type mice, the eRapa diet led to insulin resistance at 3 months (Figure 5A,B; top panels) when compared with mice on the control diet, but not at later time points of 6 or 12 months (males only) of age (Figure 5A,B; middle and bottom panels), suggesting that eRapa‐treated wild‐type mice can adapt and normalise their metabolic pathways over time.

*Pten* ^*+/−*^ mice have enhanced metabolic responsiveness, due to a prolongation of stimulated PI3K/mTORC1 signalling, with a tendency for enhanced insulin sensitivity [46, 47, 48, 49] (Figure 5A,B). eRapa diet‐fed *Pten* ^*+/−*^ mice showed no significant insulin resistance in an ITT assay when compared with mice on the control diet at 3 months for female mice (Figure 5A, top panel) and 3 and 6 months for male mice (Figure 5B, top and middle panels). eRapa‐treated female *Pten* ^*+/−*^ mice at 6 months of age and eRapa‐treated male *Pten* ^*+/−*^ mice at 1 year of age (male *Pten* ^*+/−*^ mice live longer than female mice on the control diet, allowing us to perform an additional ITT in these animals at 1 year of age), while having reduced insulin sensitivity compared with *Pten* ^*+/−*^ mice on the control diet, displayed the same insulin sensitivity as their wild‐type littermates on both the control and the eRapa diet (Figure 5A, bottom panel, and 5B, bottom panel).

GTTs of 3‐month‐old female mice showed no differences in glucose uptake in *Pten* ^*+/−*^ mice and their wild‐type littermates (Figure 5C, top panel). However, GTTs at 6 months of age in female mice (Figure 5C, right panel) and 3 and 6 months of age in males (Figure 5D, top and middle panels) revealed increased blood glucose levels in rapamycin‐treated *Pten* ^*+/−*^ mice and wild‐type littermates compared with mice on the control diet, at early time points of blood glucose measurements (15, 30, 45, and 60 min), with glucose levels returning to control levels at the later time points tested (90 min and 2 h). These observations suggest that although the dose of rapamycin used was biologically active, the amount present in the blood after 3–6 months of treatment was insufficiently high to cause sustained glucose intolerance. However, 1‐year‐old eRapa diet‐fed male mice presented with mild glucose intolerance, as indicated by increased blood glucose levels at the 90 min and 2 h time points of blood sampling compared with mice on the control diet, with the differences again being more substantial in wild‐type mice compared with *Pten* ^*+/−*^ mice (Figure 5D, bottom panel). These data again indicate that while *Pten* ^*+/−*^ mice develop some insulin resistance at an older age, they are still more adept at normalising rapamycin‐induced metabolic disruptions than their wild‐type littermates.

Consistent with the mild effects seen on insulin and glucose tolerance, fasting and fed blood glucose levels of both *Pten* ^*+/−*^ and wild‐type mice were not elevated at most time points, with the exceptions of elevated fasting blood glucose levels in rapamycin‐treated *Pten* ^*+/−*^ female mice at 3 months of age and elevated fed blood glucose levels in rapamycin‐treated *Pten* ^*+/−*^ male mice at 6 months of age, both of which appeared normalised later in life (Figure 5E,F). Another hallmark of insulin resistance is hyperinsulinaemia. In line with our findings above, insulin levels were not elevated in eRapa diet‐fed *Pten* ^*+/−*^ and wild‐type mice (Figure 5G).

In conclusion, long‐term low‐dose rapamycin treatment does not cause severe metabolic defects. With the effects of rapamycin on glucose metabolism known to be reversible [45], it could be envisaged that, in a clinical setting, treatment could be interrupted intermittently to alleviate adverse effects, or managed by standard pharmacological interventions to normalise glucose metabolism.

## Discussion

Recent advances in genomic analysis are providing increasing insight into the genetic underpinning of many rare diseases [50, 51] but also raise challenges for their prevention or treatment [52]. In a subset of rare diseases, pharmacological agents are available that in principle could correct the underlying biological defects, offering potential therapeutic opportunities. Rare diseases resulting from aberrant genetic activation of the PI3K/AKT/mTORC1/PTEN pathway are a case in point. These diseases include PHTS, TS (resulting from inactivating mutations in the mTORC1‐suppressing TSC1/2 proteins) [14, 15], *PIK3CA*‐related overgrowth syndrome (PROS; resulting from mosaic activating mutations in *PIK3CA*, the gene encoding the p110α PI3K catalytic subunit) [53], and activated PI3Kδ syndrome (APDS; due to germline activating mutations in *PIK3CD*, the gene encoding the p110δ PI3K catalytic subunit) [54] (reviewed in ref 55). Multiple PI3K/AKT/mTORC1 pathway inhibitors have been developed, mainly for cancer therapy [10], with promising clinical activity of inhibitors of PI3Kα in PROS [56] and PI3Kδ in APDS [57]. No drugs have thus far been identified that could interfere with the clinical manifestations of PHTS, an unmet clinical need that our current study has started to address.

PTEN dampens PI3K signalling by metabolising the lipids produced by the class I PI3K isoforms (PI3Kα, PI3Kβ, PI3Kγ, and PI3Kδ). Inhibitors of PI3K or their downstream targets (AKT/mTORC1) might therefore be appropriate drugs for pathologies associated with genetic PTEN defects. Here, we report a proof‐of‐concept study of cancer prevention in a mouse model of PHTS, using rapamycin to target mTORC1, a downstream effector of all class I PI3K isoforms.

PTEN inactivation by itself does not stimulate the PI3K pathway but results in ineffective PI3K pathway downregulation, leading to enhanced and prolonged signalling upon PI3K pathway activation. This also means that unstimulated PTEN mutant cells, especially in the heterozygous state, do not have a higher basal level of pAKT and downstream effectors. Based on this premise of a mild impact of heterozygous PTEN loss on the level of PI3K signalling, we reasoned that *partial* PI3K/mTORC1 pathway neutralisation might be able to neutralise the negative biological impact of systemic heterozygous PTEN inactivation. We therefore chose a low dose of rapamycin (14 ppm in food) which has previously been shown to extend lifespan in healthy mice [16, 26]. In line with published data, and as also evidenced by the modest impact on metabolic and body weight parameters monitored in our mice up to 40 weeks of age, we found that 14 ppm rapamycin in food was well tolerated in wild‐type mice, as well in *Pten* ^*+/−*^ mice. The latter is important, given that drug tolerability in the diseased/mutant state cannot be predicted from the healthy condition, as was illustrated by the observation that a low dose of rapamycin (mean plasma level of 3.4 ng/ml), which is well tolerated in other human conditions, led to substantial adverse effects in PROS patients, resulting in a high rate of discontinuation in a recent trial [58].

In the current study in the *Pten* ^*+/−*^ mouse model, we found that long‐term administration of rapamycin resulted in a highly significant extension of lifespan, accompanied by a delay in, but not full blockade of, the development of GI, thyroid, and endometrial tumours, with no impact on mammary and prostate cancer. While it is clear that the currently tested dose and dosing regimen of rapamycin is not sufficient to fully prevent cancer development, the delay by which these symptoms occur provides evidence for potential clinical benefit of long‐term rapamycin treatment in PHTS. This beneficial effect most likely derives from a dampening of PI3K/AKT/mTORC1 signalling, resulting in a prevention or delay in tumour development, rather than having a direct, acute anti‐cancer effect. Indeed, the 14 ppm dose of rapamycin used did not acutely affect the tumour growth of transplanted 4T1 syngeneic mammary tumours in wild‐type mice. That the 14 ppm dose of rapamycin was biologically active in mice is evidenced by the observations in wild‐type mice of (1) a partial reduction in mTORC1 signalling in the liver upon 4‐week dosing (Figure 1B), and (2) a reduced insulin sensitivity and glucose uptake (Figure 5A,B), both physiological hallmarks of PI3K pathway inhibition. Given the clear reduction in the grade of the lymphoid hyperplasia, a potential immunomodulatory impact may also be involved in the observed cancer‐preventative effects.

At present, the differential sensitivity of different tumour types to rapamycin is unclear. This may relate to tissue‐dependent genetic trajectories of cancer development, including changes in the tumour stroma [59], that provide distinct degrees of dependency on PI3K/mTORC1 signalling. It will be of interest to characterise the evolution of the genomic landscape of age‐related *Pten* ^+/−^ cancers in long‐term mouse studies and assess whether the beneficial impact of rapamycin on the development of some neoplasias correlates with specific genetic alterations.

To the best of our knowledge, this is the first report of a life‐long treatment of a rare disease cancer predisposition model with a PI3K/mTORC1 pathway inhibitor. The data presented here on cancer prevention by a low dose of rapamycin in a model of PHTS support the use of rapamycin, and possibly other PI3K pathway inhibitors, in a broader setting of cancer prevention, potentially as part of a so‐called ‘poly‐pill’ approach that has been shown to effectively reduce the burden of cardiovascular disease in humans [60, 61].

## Author contributions statement

PT, VR, GC and BV conceptualised the study. PT, VR, GC, WP, MA, ZV, LF, AH, GAEC, AA, SC and BV were involved in aspects of the investigation. PT, VR, GC and BV analysed data. PT, VR and BV wrote the original draft of the manuscript.

## Supporting information


Supplementary materials and methods
**Figure S1.** Tumour incidence in mice from Timed Study I
**Figure S2.** PTEN expression by immunohistochemistry in the uteri of *Pten* ^*+/−*^ female mice
**Figure S3.** PTEN and pAKT‐S473 IHC in the endometrium of mice from Timed Study II
**Figure S4.** IHC analysis of pAKT‐S473 in mammary tissues of mice from Timed Study II
**Figure S5.** Effects of long‐term rapamycin treatment on mouse body weight
**Table S1.** Reasons for euthanasia of mice
**Table S2.** Cause of illness of female mice following euthanasia at humane endpoint, based on histopathological analysis of selected tissues (GI tract, thyroid, kidney, adrenals, skin, mammary, spleen, lymph nodes, uterus, and any unusual masses)
**Table S3.** Cause of illness of male mice following euthanasia at humane endpoint, based on histopathological analysis of selected tissues (GI tract, thyroid, kidney, adrenals, skin, mammary, spleen, lymph nodes, prostate, and any unusual masses)
**Table S4.** Antibodies used for western blotting or immunohistochemistry (referred to in Supplementary materials and methods)

## Data Availability

All study data are in included in the article and/or supplementary material.
