# Supplementary material for: Long‐term treatment of cancer‐prone germline PTEN mutant mice with low‐dose rapamycin extends lifespan and delays tumour development
Source: J Pathol. 2022 Oct 31;258(4):382–94. doi: 10.1002/path.6009 (PMC9828006; doi:10.1002/path.6009)
Supplement: Supplementary file 1 — Supplementary materials and methods Figure S1. Tumour incidence in mice from Timed Study I Figure S2. PTEN expression by immunohistochemistry in the uteri of Pten +/− female mice Figure S3. PTEN and pAKT‐S473 IHC in the endometrium of mice from Timed Study II Figure S4. IHC analysis of pAKT‐S473 in mammary tissues of mice from Timed Study II Figure S5. Effects of long‐term rapamycin treatment on mouse body weight Table S1. Reasons for euthanasia of mice Table S2. Cause of illness of female mice following euthanasia at humane endpoint, based on histopathological analysis of selected tissues (GI tract, thyroid, kidney, adrenals, skin, mammary, spleen, lymph nodes, uterus, and any unusual masses) Table S3. Cause of illness of male mice following euthanasia at humane endpoint, based on histopathological analysis of selected tissues (GI tract, thyroid, kidney, adrenals, skin, mammary, spleen, lymph nodes, prostate, and any unusual masses) Table S4. Antibodies used for western blotting or immunohistochemistry (referred to in Supplementary materials and methods) [file PATH-258-382-s001.docx]

**Long-term treatment with low-dose rapamycin extends lifespan and delays tumours in cancer-prone germline PTEN mutant mice**

P Tibarewal *et al. J Pathol* <https://doi.org/10.1002/path.6009>

**Supplementary materials and methods**

**Supplementary Figures S1–S5**

**Supplementary Tables S1–S4**

**Supplementary materials and methods**

Reference numbers refer to the main text list

*Mouse genotyping*

Genotyping of mice was performed using tissues from ear biopsies. The tissue was lysed in a buffer containing 25 mm NaOH/0.2 mm disodium EDTA at 95 °C for 1 h, followed by neutralisation with an equal volume of a buffer containing 40 mm Tris–HCl, pH 4.5. PCR reactions were performed with the DNA extract using Titanium Taq polymerase (Clontech, Shiga, Japan) following the manufacturer’s protocol and PCR products run on an agarose gel. The PCR primers used were Pten common TTGCACAGTATCCTTTTGAAG (100 nm), Pten WT GTCTCTGGTCCTTACTTCC (50 nm), and Pten Neo ACGAGACTAGTGAGACGTGC (50 nm), with the following PCR conditions: 95 °C for 1 min, 30 cycles of 95 °C for 30 s, 60 °C for 30 s, 68 °C for 30 s, followed by a cycle of 68 °C for 7 min. DNA from wild-type mice resulted in a single PCR product of 240 bp, with *Pten^+/−^* mice giving an additional PCR product of 320 bp.

*Mouse tissue histology*

Mouse tissues were harvested after euthanasia and fixed in 10% formalin for 24 h and then processed and embedded into paraffin wax blocks. The organs were orientated with the largest surface down, to obtain complete sections, and 3-µm serial sections were cut and stained using haematoxylin and eosin. Slides were scanned using a NanoZoomer S360 (Hamamatsu Photonics UK, Welwyn Garden City, UK), and photomicrographs were analysed using ImageJ (<https://imagej.nih.gov/ij/>) or NDPView2 (Hamamatsu).

*4T1 experiments*

BALB/c mice were purchased from Charles Rivers Laboratories (Boston, MA, USA). The 4T1 mammary cancer cell line was purchased from the ATCC (Manassas, VA, USA) and cultured in RPMI culture medium containing 10% FBS: 100 µl of a single cell suspension containing 25,000 4T1 cells was injected subcutaneously into the right flank of 8- to 10-week-old female BALB/c mice. On day 6 post-injection, the mice were switched to the control diet or the eRapa diet or given an i.p. injection of rapamycin at 8 mg/kg or vehicle for 5 days, with no injections on weekends (Saturday/Sunday). For rapamycin injection, rapamycin (SelleckChem, Houston, TX, USA) was dissolved in DMSO to 100 mg/ml, then further diluted in 5% PEG-400–5% Tween-80 to a final concentration of 0.8 mg/ml and filtered through a 0.22 µm filter. Tumour length and width were measured using callipers, and tumour volume was calculated using the formula short measurement × long measurement^2^ × 0.52. Mice were sacrificed on day 26. Liver tissues were snap-frozen in liquid nitrogen for western blot analysis.

*Analysis of rapamycin concentrations in mouse plasma*

Blood samples were collected from mice via cardiac puncture under terminal anaesthesia. Plasma was collected by centrifuging the blood at 5000 × *g* for 10 min at 4 °C: 50 µl of standard or sample was mixed with 10 µl of internal standard working solution (100 ng/ml erythromycin in water) and 500 µl of tert-butyl methyl ether was added, followed by sonication in an ultrasonic bath (3 × 10 min) and centrifugation. The top layer was removed, dried down, and reconstituted in 50 µl of 1:1 methanol–15 mm ammonium formate, pH 3.5, v/v. Separation was performed on an ACE C18 3 µm, 2.1 × 100 mm column using a Waters Acquity UHPLC system (Waters, Kowloon, Hong Kong SAR, PR China). Mobile phase A was 15 mm ammonium formate, pH 3.5 and mobile phase B was methanol with a gradient of 50–100% B 0–2 min, hold at 100% B 2–5 min, returning to 50% B at 5.1–8 min with a column temperature of 60 °C and a flow rate of 0.3 ml/min. Analysis was in ES+ mode detecting SIR rapamycin *m/z* 936.6 and erythromycin *m/z* 734.4 using a Waters TQD mass spectrometer.

*Insulin tolerance tests* (*ITTs*) *and glucose tolerance tests* (*GTTs*)

Mice were starved for 6 h. The tip of the tail was punctured using a 26G needle and time-0 blood glucose levels were recorded using a standard glucometer. For ITTs, mice were given an i.p. injection of insulin to a final concentration of 0.75 U/kg for male mice and 0.5 U/kg for female mice. For GTTs, the mice were given an i.p. injection of glucose to a final dose of 2 g/kg. Blood glucose readings were recorded at 15, 30, 45, 60, 90, and 120 min post-injection.

*Insulin ELISA*

Mouse blood was harvested at 6 months of age at 9 am. The blood was centrifuged at 5000 × *g* for 10 min at 4 °C. The plasma was collected and stored at −80 °C. Plasma insulin concentrations were measured using an ELISA kit (CrystalChem, Elk Grove Village, IL, USA) following the manufacturer’s instructions.

*Western blot analysis*

Mice were euthanised and tissues harvested and snap-frozen in liquid nitrogen and stored at −80 °C. Liver tissue was homogenised using Lysing Matrix M tubes (MP Biomedicals, Santa Ana, CA, USA) in double the volume (v/w) of lysis buffer [25 mm Tris–HCl (pH 7.4), 150 mm NaCl, 1% Triton X-100, 0.1% SDS, 10% glycerol, 1 mm EGTA, 1 mM EDTA, 10 mm sodium pyrophosphate, 20 mm β-glycerophosphate, 100 mm sodium fluoride, 2 mm sodium orthovanadate, 1 mm DTT and protease inhibitors] on a FastPrep 24 homogeniser (MP Biomedicals) at 4 m/s for 20 s. The homogenate was pre-cleared by centrifugation at 20,000 × *g* for 10 min and the protein extracts were analysed by immunoblotting. Protein gel electrophoresis was conducted with 50 µg of total soluble protein per sample using NuPage Bis-Tris 4–12% gradient polyacrylamide gels (Thermo Fisher Scientific, Waltham, MA, USA) following the manufacturer’s protocols. Proteins were transferred onto PVDF membrane (Millipore, Burlington, MA, USA) and membranes blocked in 5% milk powder/TBST for 1 h at room temperature. Blocked membranes were incubated overnight with primary antibodies. Details of the antibodies and dilutions used can be found in supplementary material, Table S4. Antibody complexes were detected by 1 h incubation at room temperature with HRP-conjugated secondary antibodies (GE Healthcare, Chicago, IL, USA). Immunoblots were developed with Immobilon Forte Western HRP substrate (Millipore) and imaged directly using an ImageQuant LAS4000 imaging system (GE Healthcare) and analysed using ImageQuantTL software (GE Healthcare).

*Mouse tissue immunohistochemistry*

Immunohistochemistry was performed using a Leica BOND RXm automated processor (Leica, Wetzlar, Germany). Tissue sections were incubated at 100 °C in BOND epitope retrieval solution 2 (Leica), followed by blocking and staining using the BOND polymer refine detection kit (Leica, DS9800), then primary antibody (supplementary material, Table S4) and DAB enhancer (Leica).

*Histopathological analysis*

Non-neoplastic lesions, e.g. splenic extramedullary haematopoiesis and lymph node hyperplasia, were graded using a standard semi-quantitative grading scheme from 0 to 5, where 0 = lesion not present and 5 = the maximum lesion size/extent [62]. In general, where present, the presence of other proliferative lesions including hyperplasia and neoplasia was recorded for each individual organ, allowing the overall incidence to be established for each lesion in each group. In addition, specific scoring protocols were used for endometrial hyperplasia and PIN. The Milam scoring methodology [32] was used for endometrial hyperplasia, which was categorised using the following system according to the percentage (score 1, <33% of hyperplastic lesion; score 2, 33–66%; score 3, >66%) and the size of the hyperplastic lesions (score 1, the hyperplastic glands were small and regular; score 2, mix of small and large size; score 3, more than half of the hyperplastic glands were large and irregular). The two scores were then multiplied. The hyperplastic lesion was defined as grade 1 when the product of the two scores was 1–3, as grade 2 when the product was 4–6, and as grade 3 when the product was 7–9. Prostatic intraepithelial neoplasia (PIN) was graded as low or high grade according to the consensus suggested by Ittmann *et al* [63]. Low-grade PIN lesions are focal, with one to two layers of cells and mild nuclear atypia. High-grade lesions tend to be more extensive, filling the prostatic lumen but without stromal invasion; have two or more cell layers often forming papillary or cribriform patterns; increased nuclear atypia and mitoses.

*Statistics and sample size calculation for life-long rapamycin study*

These calculations were performed by Katrina Gore from KDL Gore Solutions Ltd, UK. We used 𝜒^2^ or Fisher’s exact test, with a desired statistical power of 80% and significance level of 5% for sample size calculations. Assuming that the proportion of animals on the control diet with tumours was 95% and that the proportion of animals on the eRapa diet with tumours was 65%, the required number of mice in each group was calculated to be 33. The statistical tests used for individual datasets have been described in the figure legends.

**Supplementary Figures S1–S5**

**
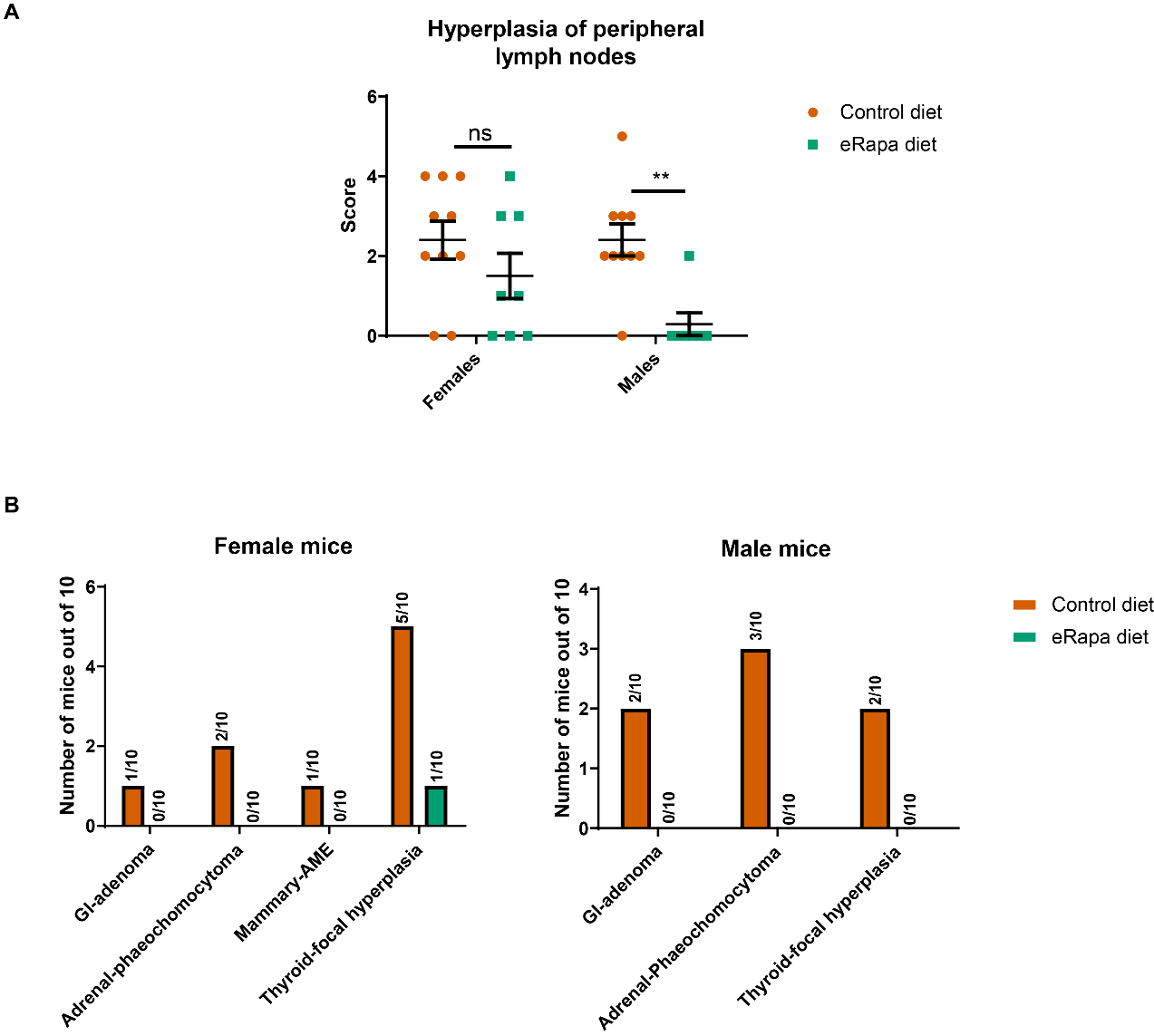
**

**Figure S1.** **Tumour incidence in mice from Timed Study I.** *Pten^+/−^* mice on a mixed (C57BL/6J x SV129) background were fed the control or eRapa diet from the age of 6 weeks onwards and euthanised at 6 months of age. (A) Grade of lymphoid hyperplasia in *Pten^+/−^* mice on the control or eRapa diet. (B) Incidence of tumours in female (left panel) and male (right panel) *Pten^+/−^* mice on the control or eRapa diet.

**
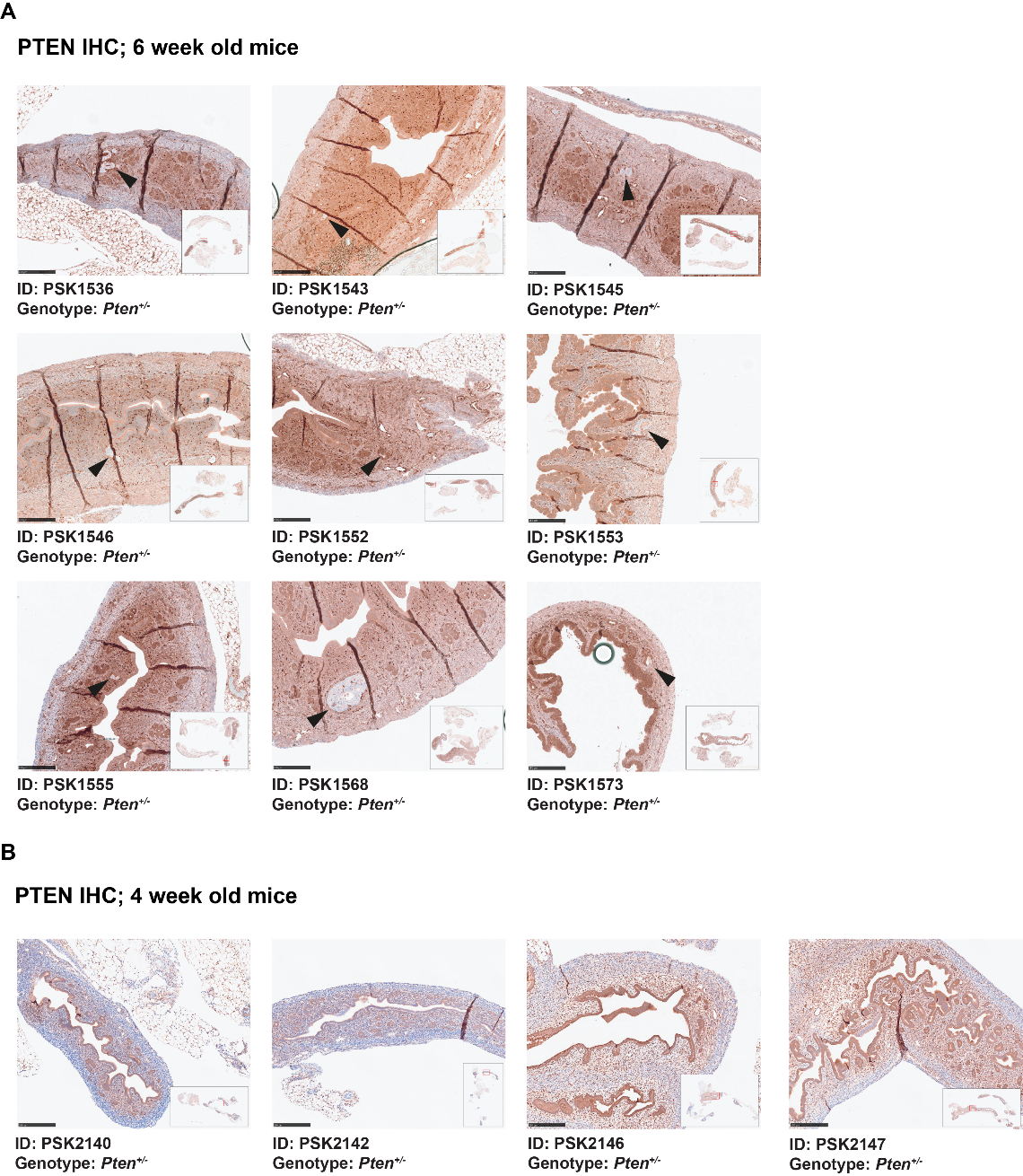
**

**Figure S2.** **PTEN expression by immunohistochemistry in the uteri of *Pten^+/−^* female mice.** Additional images of IHC performed on uteri from 4- and 6-week-old female *Pten^+/+^* and *Pten^+/−^* mice showing loss of PTEN immunoreactivity in endometrial glands of *Pten^+/−^* mice at 6 weeks (indicated by black arrowheads) but not at 4 weeks of age. The inset shows the entire tissue with the area of PTEN loss magnified in the main image. A random area was magnified in the image from 4-week-old mice. Scale bars represent 250 µm. This is an extension of data in Figure 3E.


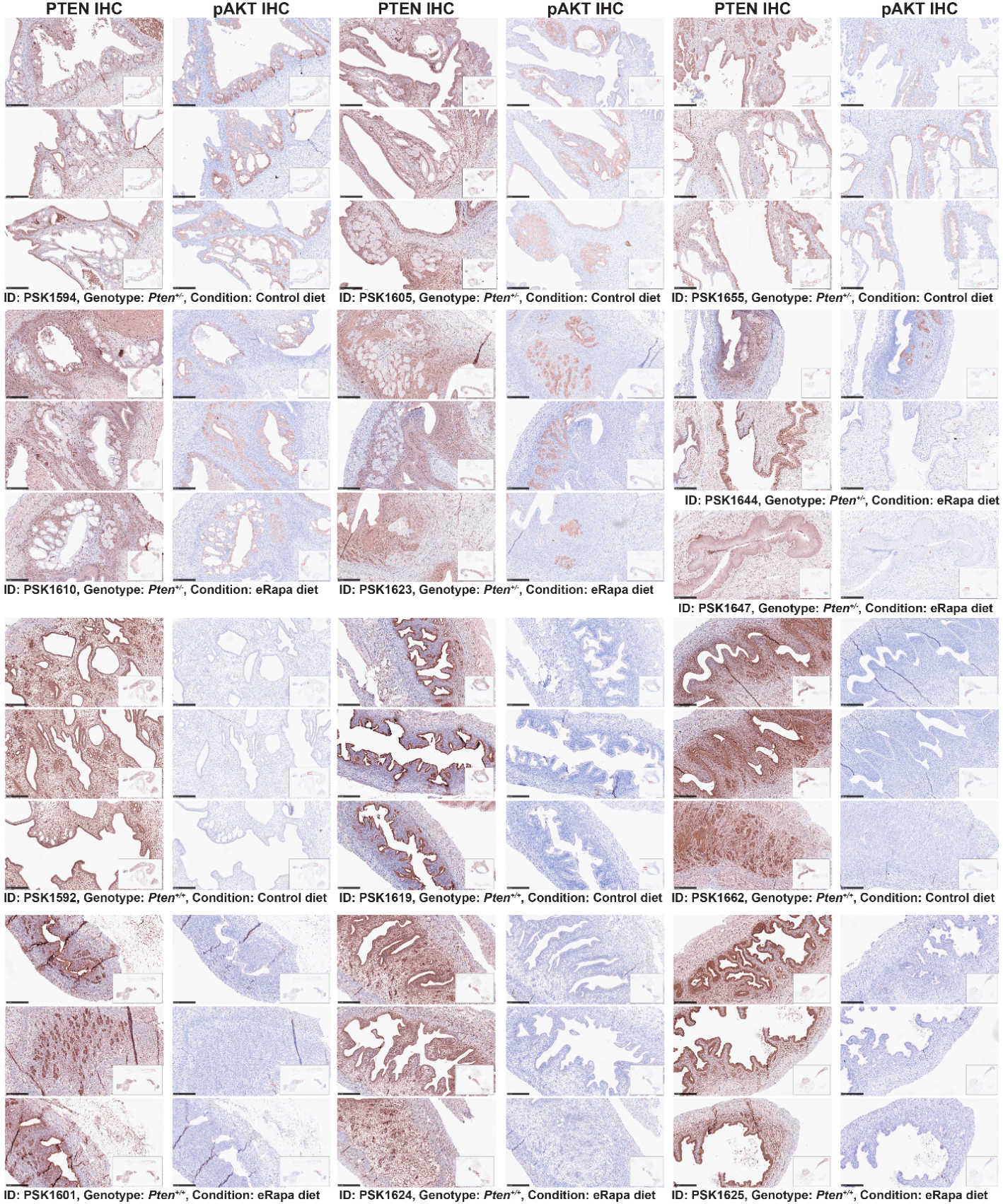


**Figure S3.** **PTEN and pAKT-S473 IHC in the endometrium of mice from Timed Study II.** Female *Pten^+/−^* mice and littermate *Pten^+/+^* mice were fed the control or eRapa diet from 4 weeks of age and sacrificed at 6 months of age. Additional examples of IHC performed on uteri showing loss of PTEN immunoreactivity in endometrial glands of *Pten^+/−^* mice on the control or eRapa diet, with a corresponding increase in pAKT-Ser473 immunoreactivity within the same glands. The inset shows the entire tissue with three random areas magnified in the main image. Scale bars represent 250 µm. This is an extension of data in Figure 3F.

**
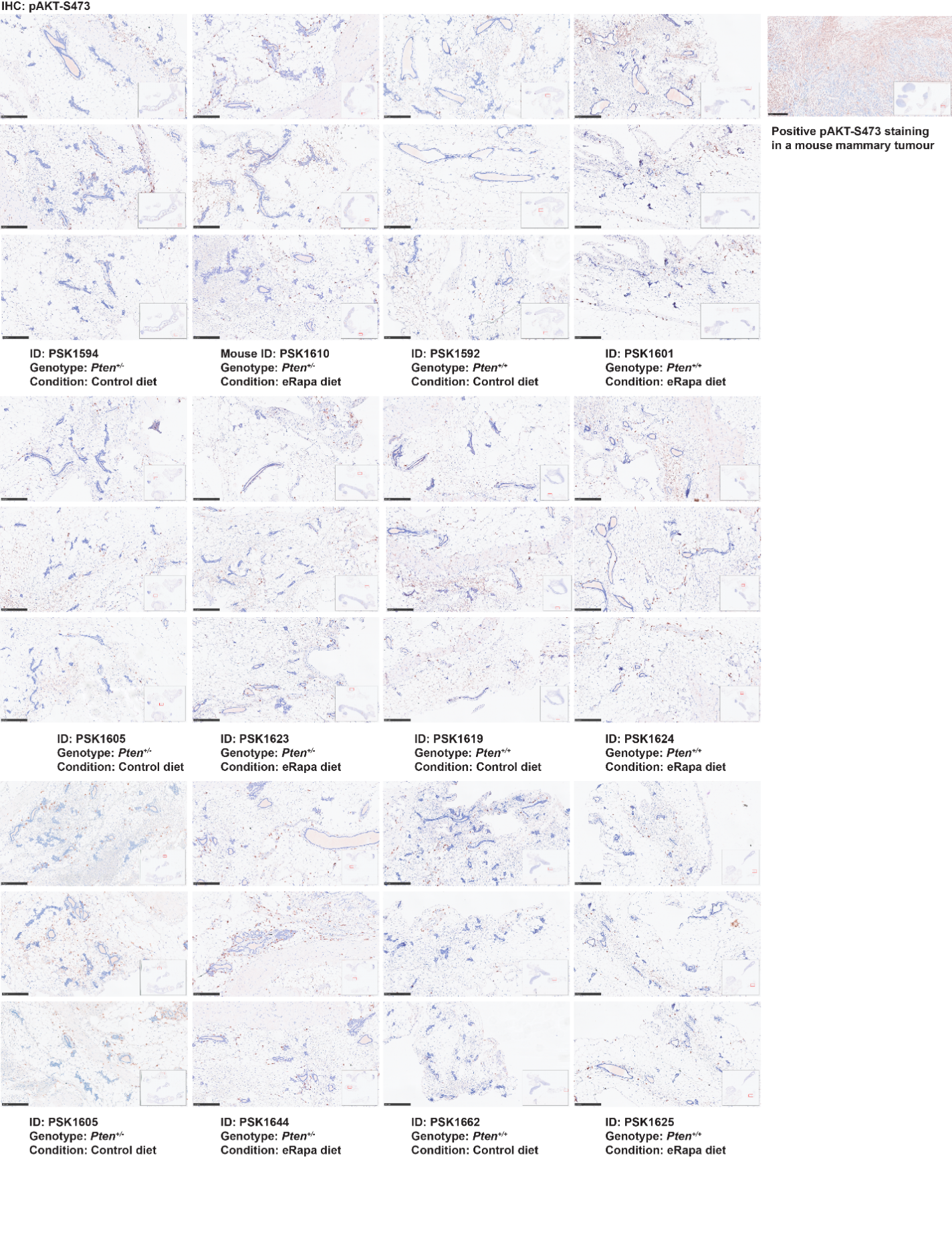
**

**Figure S4.** **IHC analysis of pAKT-S473 in mammary tissues of mice from Timed study II.** Female *Pten^+/−^* mice and littermate *Pten^+/+^* mice were fed the control or eRapa diet from 4 weeks of age and sacrificed at 6 months of age. Additional examples of IHC performed for pAKT-S473 on mammary tissue. The inset shows the entire tissue with three random areas magnified in the main image. No signal for pAKT-S473 was observed in mammary tissues of mice on the control or eRapa diet. Staining for pAKT-S473 in endometrial tissue of the same mouse processed on the same slide can be seen in the inset. Note the positive pAKT-S473 staining in *Pten^+/−^* endometrium but absence of the staining in *Pten^+/+^* endometrium. Also shown is positive staining for pAKT-S473 in a mouse mammary tumour sample as an additional positive control. Scale bars represent 250 µm.


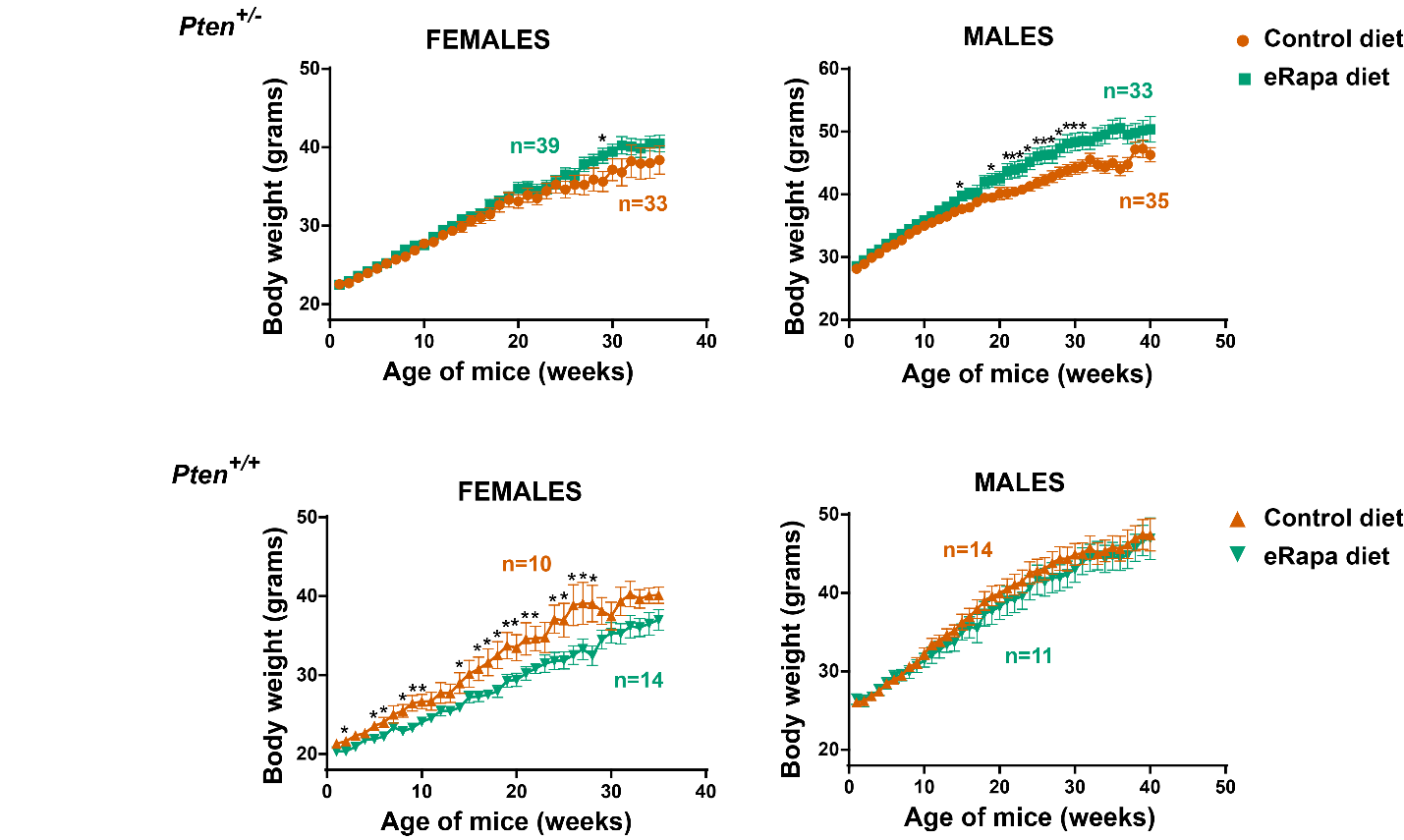


**Figure S5.** **Effects of long-term rapamycin treatment on mouse body weight.** *Pten^+/−^* mice and littermate *Pten^+/+^* mice were fed the control or eRapa diet from 6 weeks of age. Body weight of mice over the indicated time points. Statistical analysis was performed using the unpaired *t*-test with Welch’s correction. **p* < 0.05; ***p* < 0.01; ****p* < 0.001; *****p* < 0.0001.

**Supplementary Tables S1–S4**

|  | **Control diet** | | | | **eRapa diet** | | | | |
| --- | --- | --- | --- | --- | --- | --- | --- | --- | --- |
| **Reason for euthanasia** | **Lumps** | **Sickness** | **Both lumps & sickness** | **Found dead** | **Lumps** | **Sickness** | **Both lumps & sickness** | **Found dead** | **End of study** |
| Female | 23/33  70% | 6/33  18% | 4/33  12% | 0/33  0% | 27/38  71% | 9/38  24% | 2/38  5% | 0/38  0% | 0/38  0% |
| Male | 18/32  56% | 8/32  25% | 1/32  3% | 5/32  16% | 15/34  44% | 13/34  38% | 1/34  3% | 1/34  3% | 4/34  12% |

**Table S1.** Reasons for euthanasia of mice.

**Table S2**. Cause of illness of female mice following euthanasia at humane endpoint, based on histopathological analysis of selected tissues (GI tract, thyroid, kidney, adrenals, skin, mammary, spleen, lymph nodes, uterus, and any unusual masses).

| **Pathological finding** | **Control diet** | **eRapa diet** |
| --- | --- | --- |
| Lymphoid hyperplasia | 64% (21/33) | 16% (6/38) |
| Lymphoma | 3% (1/33) | 3% (1/38) |
| Glomerulonephropathy | 12% (4/33) | 13% (5/38) |
| Mammary tumour | 12% (4/33) | 53% (20/38) |
| Uterine haemorrhage | 3% (1/33) | 5% (2/38) |
| Endometritis/pyometra/abscess | – | 3% (1/38) |
| Histiocytic sarcoma | – | 3% (1/38) |
| Uncertain from submitted slides | 15% (5/33) | 5% (2/38) |

**Table S3.** Cause of illness of male mice following euthanasia at humane endpoint, based on histopathological analysis of selected tissues (GI tract, thyroid, kidney, adrenals, skin, mammary, spleen, lymph nodes, prostate, and any unusual masses).

| **Pathological finding** | **Control diet** | **eRapa diet** |
| --- | --- | --- |
| Lymphoid hyperplasia | 56% (18/32) | 38 (13/34) |
| Lymphoma | – | 9% (3/34) |
| Preputial gland tumour | – | 6% (2/34) |
| Haemangiosarcoma | 63% (2/32) | 3% (1/34) |
| Urinary tract obstruction | 93% (3/32) | – |
| Liver tumour | – | 12% (4/34) |
| Uncertain from submitted slides | 25% (8/32) | 20% (7/34) |
| End of study | – | 12% (4/34) |

**Table S4.** Antibodies used for western blotting or immunohistochemistry.

| **Antibody for western blotting** | **Supplier** | **Catalogue number** | **Species raised in** | **Dilution** |
| --- | --- | --- | --- | --- |
| pS6 Ser240/244 | Cell Signaling Technology, Danvers, MA, USA | 2215 | Rabbit | 1:1,000 |
| Total-S6 | Cell Signaling Technology | 2217 | Rabbit | 1:1,000 |
| Vinculin | Sigma Aldrich, St Louis, MO, USA | V-9131 | Mouse | 1:5,000 |

| **Antibody for immunohistochemistry** | **Supplier** | **Catalogue number** | **Species raised in** | **Dilution** |
| --- | --- | --- | --- | --- |
| PTEN | Cell Signaling Technology | 9559 | Rabbit | 1:100 |
| pAKT Ser473 | Cell Signaling Technology | 4060 | Rabbit | 1:50 |
